# The diagnostic utility of microscopic quality assessment of sputum samples in the era of rapid syndromic PCR testing

**DOI:** 10.1128/spectrum.03002-23

**Published:** 2023-09-29

**Authors:** Dagfinn Lunde Markussen, Marit Ebbesen, Sondre Serigstad, Siri Tandberg Knoop, Christian Ritz, Rune Bjørneklett, Øyvind Kommedal, Synne Jenum, Elling Ulvestad, Harleen M. S. Grewal

**Affiliations:** 1 Emergency Care Clinic, Haukeland University Hospital, Bergen, Norway; 2 Department of Clinical Science, Bergen Integrated Diagnostic Stewardship Cluster, Faculty of Medicine, University of Bergen, Bergen, Norway; 3 Department of Microbiology, Haukeland University Hospital, Bergen, Norway; 4 Department of Clinical Medicine, Faculty of Medicine, University of Bergen, Bergen, Norway; 5 National Institute of Public Health, University of Southern Denmark, Copenhagen, Denmark; 6 Department of Infectious Diseases, Oslo University Hospital, Oslo, Norway; University of Maryland School of Medicine, Baltimore, Maryland, USA

**Keywords:** pneumonia, diagnostics, rapid tests, quality assurance, molecular methods, sputum, microcopy, gram stain, syndromic PCR, filmarray

## Abstract

This prospective study assessed the value of initial microscopy evaluation of sputum samples submitted for rapid syndromic PCR-based testing. Bacterial detections by the BioFire FilmArray Pneumonia Panel *plus* in 126 high- and 108 low-quality sputum samples, based on initial microscopy evaluation in samples from patients with lower respiratory tract infections were compared. We found that high-quality samples had a higher proportion of bacterial detections compared to low-quality samples (*P* = 0.013). This included a higher proportion of detections of bacteria deemed clinically relevant by predefined criteria (70% and 55%, *P* = 0.016), as well as a higher proportion of detections of *Haemophilus influenzae* (36% and 20%, *P* = 0.010). High-quality samples also had more detections of bacteria with high semi-quantitative values. The study found no significant difference between high- and low-quality samples in the proportions of samples with a single species of bacteria detected, samples with a bacteria treated by the clinician, samples with detection of a proven etiology of community-acquired pneumonia by predefined criteria, the number of bacterial species detected, or the detection of *Streptococcus pneumoniae*, *Moraxella catarrhalis*, or *Staphylococcus aureus*. The results showed that 40% (95% CI 35%–47%) of the bacterial detections would have been missed if only high-quality samples were analyzed. This included 41% (27%–56%) of detections of *S. pneumoniae*, 33% (23%–45%) of detections of *H. influenzae*, 42% (28%–58%) of detections of *S. aureus*, and 37% (23%–54%) of detections of *M. catarrhalis*. These findings suggest that all sputum samples submitted for rapid syndromic PCR testing should be analyzed, regardless of initial microscopy quality assessment. (This study has been registered at ClinicalTrials.gov under registration no. NCT04660084.)

**IMPORTANCE:**

Microscopic quality assessment of sputum samples was originally designed for sputum culture, and its applicability in today’s workflow, which includes syndromic PCR testing, may differ. Addressing this crucial gap, our study emphasizes the need to optimize the use and workflow of syndromic PCR panels, like the BioFire FilmArray Pneumonia plus (FAP plus), in microbiology laboratories. These advanced PCR-based tests offer rapid and comprehensive pathogen detection for respiratory infections, yet their full potential remains uncertain. By comparing bacterial detections in high- and low-quality sputum samples, we underscore the importance of including low-quality samples in testing. Our findings reveal a significant proportion of potentially clinically relevant bacterial detections that would have been missed if only high-quality samples were analyzed. These insights support the efficient implementation of syndromic PCR panels, ultimately enhancing patient care and outcomes.

## INTRODUCTION

The microbiologic diagnosis of community-acquired pneumonia (CAP) is challenging, and the value of sputum culture in determining the etiology is a topic of debate (1). Pathobionts from the oropharyngeal flora can contaminate sputum samples and falsely be interpreted as causative agents and/or the oropharyngeal flora can overgrow true pathogens thereby giving false negative results. The current guidelines recommend that only sputum samples judged to be representative of the lower respiratory tract (LRT) by predefined microscopy criteria of Gram-stained samples are cultured, leading to no further processing of specimens with inadequate quality (2). However, the utility of the Gram stain evaluation has been questioned in several studies, as sensitivity, specificity, interrater agreement, and accuracy vary substantially between different settings (3 – 5). The microscopy criteria in use are diverse (6), but the most widely used criteria for determining the quality of sputum samples were proposed Bartlett in 1974. As clearly stated by Bartlett, the criteria were not based on clinical evidence but mainly motivated by cost and labor reduction in the laboratory (7). A recent study on pneumococcal etiology in CAP reports on the value of performing standard culture of expectorated sputum irrespective of prior quality assessment by microscopy (8).

When the microscopy criteria for the evaluation of sputum quality were developed, the LRT in healthy lungs was thought to be sterile. However, it is now increasingly accepted that healthy lungs in general harbor a dynamic microbiome likely dependent on host factors like chronic lung disease and intercurrent infections. The correlation between the oral and lung microbiome is high. There is an increasing understanding of the respiratory microbiota as a continuity that interacts through the airways but with decreasing numbers of microbes toward the lower airways and alveoli (9 – 11). Syndromic PCR panels can detect and quantify potentially pathogenic microbes despite a complex and diverse background microbiome. Additional advantages over traditional culture-based methods are the ability to detect non-culturable microbes, the detection of bacteria despite the administration of antibiotics prior to sampling, and the detection of resistance genes. Some syndromic panels also provide semi-quantitative results with cut-offs to prevent reporting of microorganisms detected in low quantities (12, 13). The status of culture as a gold standard for LRT infections is being challenged, considering several limiting factors including prior receipt of antibiotics, poor growth of fastidious bacteria, and subjective interpretation of culture results (14).

The study will compare bacterial detections by rapid syndromic PCR-based testing in high- and low-quality sputum samples, based on the initial microscopy evaluation, and determine the proportion of detections that would have been missed if only high-quality samples were analyzed. To our knowledge, no previous studies have evaluated the role of microscopy quality assessment of sputum samples prior to comprehensive rapid molecular microbiological diagnosis of CAP in adults.

## MATERIALS AND METHODS

### Study setting

This investigation was nested within a randomized controlled trial (RCT; NCT04660084) conducted at Haukeland University Hospital, a tertiary care referral center in Bergen, Norway.

### Study participants

Patients were eligible for inclusion if they were ≥18  years, presenting to the emergency department (ED) with a suspicion of CAP (evaluated by investigating physicians and/or study nurses). The details of the inclusion criteria in the RCT and study procedures have been described previously (15, 16). All patients who provided sputum sample and had a confirmed respiratory tract infection (RTI) were included in the analysis.

### Sample collection

Nebulized saline solution (isotonic or hypertonic) was given to all patients. If patients were able to produce a visually more purulent spontaneous sputum sample than that obtained by saline induction, the former was sent for further testing. Study nurses or doctors supervised all sputum collection and graded each sample according to a validated visual sputum chart (17). Data about past medical history and co-morbidities were obtained from electronic medical journals (15).

### Microscopy evaluation

Microscopy was performed on sputum samples from all patients according to guidelines; a purulent-looking portion was Gram stained, and samples with <10 squamous epithelial cells (SECs) or a ratio of leukocytes/SECs ≥10 and >5 microbes per field at a 100×  magnification, were classified as high quality (2). All other samples were classified as low quality.

### Rapid syndromic PCR-based testing

The real-time multiplex PCR panel, the BioFire FilmArray Pneumonia Panel *plus* (bioMérieux, France) (FAP *plus*), was applied on sputum samples in addition to standard-of-care methods. These included culture of respiratory tract samples and blood, nasopharyngeal and/or oropharyngeal swabs analyzed by an in-house real-time PCR test for detection of respiratory viruses (SARS CoV-2, influenza A and B, human parainfluenza viruses 1–3, respiratory syncytial virus, human metapneumovirus, and rhinovirus) and atypical bacteria (*Bordetella pertussis*, *Bordetella parapertussis*, *Mycoplasma pneumoniae*, and *Chlamydia pneumoniae*), rapid pneumococcal urine antigen test, and any additional tests requested by the treating physician. The FAP *plus* integrates nucleic acid extraction, reverse transcription, and nested multiplex PCR amplification for 9 viruses, 18 bacteria, and 7 antimicrobial resistance genes. The device is intended for use with sputum-like specimens (expectorated or induced sputum and endotracheal aspirates) and bronchoalveolar lavage specimens tested directly, without pretreatment. In addition to nucleic acid detection, the panel is able to provide a semi-quantitative estimate of abundance for 15 of the bacterial targets (reported in log_10_ increments from 10^4^ to 10^7^ genomic copies/mL). All testing is done in the closed sample-to-answer FilmArray system, which provides automated analysis and results in about 75 min. As part of the RCT design, LRT samples were randomized to immediate or delayed testing with the FAP *plus* where results from the latter were not made available to the treating physician.

### Culture

Both high- and low-quality samples were cultured with semi-quantitative measurement of growth using a scale of 0 to 4+. For the culture of sputum samples, *Streptococcus pneumoniae*, *Haemophilus influenzae*, *Moraxella catarrhalis*, and beta-hemolytic streptococci group A, B, C, and G were reported if detected in pure culture or dominating in a mixed culture. Other bacterial detections such as Enterobacterales and *Staphylococcus aureus* were only reported if clinical information provided in the microbiology lab requisition form suggested underlying disease with increased risk of pneumonia such as chronic pulmonary disease, recent antibiotic use, or immunosuppression. Mixed respiratory flora was reported in cultures where no single microbe dominated or which were dominated by low virulent bacteria such as viridans streptococci or coagulase-negative staphylococci.

### Diagnostic classification of respiratory infections

Final clinical diagnoses were determined retrospectively based on pre-specified diagnostic criteria (16). Patients were classified into five groups: CAP, non-infectious exacerbation of chronic obstructive pulmonary disease (COPD), infectious exacerbation of COPD, other RTIs, and other diagnoses. CAP and infectious exacerbation of COPD were considered LRT infections. The definition of CAP was adapted from Postma and colleagues (18). For a patient to be classified as having CAP, there had to be documentation of in-hospital treatment and/or a diagnosis of clinically suspected CAP that was in agreement with the assessment of two study physicians. In cases where there was disagreement between the two physicians, a third physician who was not study-related served as an arbitrator. Additionally, there had to be at least two clinical criteria, including the presence of a new or increased infiltrate on chest radiography or computer tomography as documented in the written radiology report. Infectious COPD exacerbations were defined as documentation of in-hospital treatment and/or a diagnosis of infectious COPD exacerbation with at least two clinical criteria and not meeting the criteria for CAP.

### Assignment of microbiological etiology

There is no current gold standard for establishing the microbiologic etiology of CAP. We used a pragmatic approach and based our etiological assignment on a combination of methods used in other studies. These included the detection of a single bacterial species, the detection of bacteria deemed clinically relevant by the treating physician, the detection of mixed respiratory flora, and the quantification of potentially pathogenic bacteria (12, 14, 19 – 24).

Bacterial detections were retrospectively classified according to pre-specified criteria, regardless of whether the patients were in the group where the FilmArray Plus results were reported or not.

Bacterial detections were classified as proven, probable, or uncertain etiological causes of LRTI. Proven etiology was defined as detection of the same bacterial species in blood cultures and in a respiratory tract sample, or *S. pneumoniae* detected in respiratory tract specimen AND a positive pneumococcal urine antigen test, or *Legionella pneumophilia* detected in a respiratory tract sample AND a positive urine antigen test in a patient with an LRTI.

To classify probable etiology, the targets for the FilmArray pneumo plus panel were grouped into different categories based on their potential clinical relevance as per the current literature on CAP. Category A included pathogens that are always considered relevant in patients with community-aquired pneumonia, such as most viral detections, detection of *B. pertussis*, *C. pneumoniae*, *L. pneumophila*, and *M. pneumoniae*. Category B comprised usually pathogenic bacteria, including *H. influenzae*, *S. pneumoniae*, and *Streptococcus pyogenes*, which can sometimes be colonizers but are usually considered relevant in pneumonia. Category C included usually non-pathogenic bacteria, such as *M. catarrhalis*, *Klebsiella pneumoniae*, and *S. aureus*, which are usually colonizers but can cause pneumonia, especially in patients with chronic underlying diseases. Category D included usually non-pathogenic bacteria, such as *Pseudomonas aeruginosa*, Enterobactrales, and *Acinetobacter* spp, which are a rare cause of pneumonia in healthy patients but may be considered relevant in immunocompromised patients or patients with a recent history of antibiotic use. A probable etiology was defined as a diagnosed pneumonia and the detection of one or more of the following criteria: (i) category B-bacteria in respiratory tract specimen; (ii) category C-bacterium in respiratory tract specimen in patients with chronic underlying diseases and/or a recent history of antibiotic use; (iii) detection of one species of category D-bacteria as the only bacterial species identified in respiratory tract specimen in immunocompromised patients and/or patients with a recent history of antibiotic use or other known risk of infections due to certain bacteria; (iv) detection of category D-bacterium in blood culture and no other possible focus of infection; and (v) positive pneumococcal urine antigen test. More details on the diagnostic classification have been previously published (16).

A bacteria deemed clinically relevant by the treating physician was defined as a detection that was documented as relevant by the treating physician in the electronic health record and/or resulted in a change in the antimicrobial therapy. Bacterial detections in patients with other RTIs than CAP and infectious exacerbation of COPD were not analyzed as national guidelines do not recommend antimicrobial treatment for these conditions (25).

### Statistics

For the primary analysis of patient characteristics and clinical findings, we included all patients that provided a sputum sample. For the analysis of microbiologic detections, we included only patients with a confirmed LRT infection at discharge, including CAP and infectious exacerbation of COPD.

Categorical data were summarized as counts and percentages of the total, and differences in proportions were analyzed by χ^2^ test for expected values above 10 and by Fisher’s exact test for expected values of 10 or below. Continuous variables were summarized as medians with interquartile range (IQR) and compared by Mann-Withney-U test. Not normally distributed variables were log-transformed. A two-tailed *P*-value  ≤  0.05 was considered statistically significant for all analyses. Two-sided 95% confidence intervals for proportions were computed by the modified Wald method.

Statistical analyses were performed using IBM SPSS Statistics (Statistical Package for the Social Sciences) version 26.0 (IBM Corp, Armonk, NY, USA) and GraphPad QuickCalcs website: https://www.graphpad.com/quickcalcs/ (last accessed on 26 January 2023).

## RESULTS

**Fig 1:**
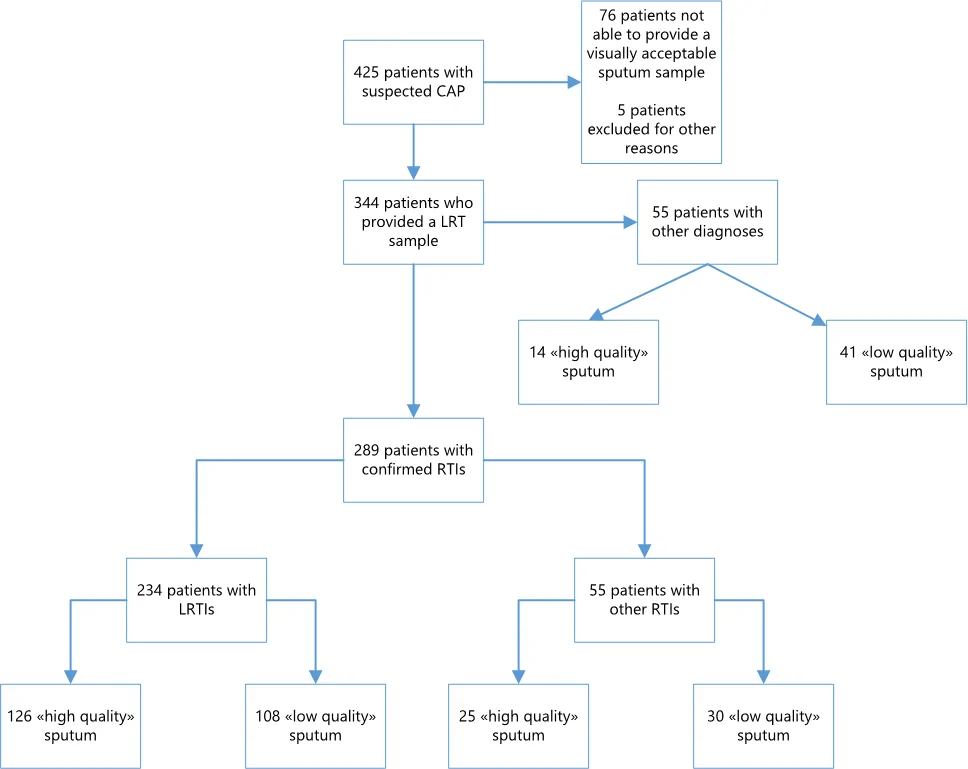
Flow chart of patients included in the study abbreviations: CAP, community-acquired pneumonia; LRT, lower respiratory tract; RTI, respiratory tract infection; LRTI, lower respiratory tract infection.

Between 25 September 2020 and 21 June 2022, a total of 425 patients with suspected CAP were included in the study. A flow chart of patients included in the study is shown in Fig. 1. Of the 425, 66 (15.5%) patients were unable to provide a visually mucoid or purulent LRT sample, 10 (3.3%) provided endotracheal aspirates for analysis and were not included in the analysis, and 5 (1.2%) were excluded for other reasons, and 55 (12.9%) were diagnosed with conditions other than RTI infection. The final study cohort consisted of 289 patients, of which 234 (80.9%) were confirmed to have an LRT infection, and 55 (19.1%) had other RTIs. In total, 151 (52.2%) samples were classified as high-quality by microscopy. According to the randomization process in the RCT, FAP *plus* was performed immediately after testing for 139 (48.1%) samples, and for 150 (51.9%) samples, delayed testing was performed, with the results not made available to the treating physician. Demographic and clinical characteristics of the patients with RTIs are presented in Table 1. More patients randomized to delayed FAP *plus* testing had a high-quality sample, 89 (57.8%) vs 66 (45.5%), *P* = 0.023. For patients with LRT infection, there was no significant difference in the proportion of patients randomized to immediate or delayed FAP *plus* testing between patients with high- and low-quality sputum samples (*P* = 0.075). Patients with high-quality sputum samples more frequently had COPD in their medical history, and higher white blood cell counts and C reactive protein (CRP) levels than patients with low-quality samples.

**TABLE 1:** Patient characteristics for patients with respiratory tract infections (*n* = 289)^*d*^

	High-quality sputum^*b*^ *n* = 151	Low-quality sputum^*b*^ *n* = 138	*P*-value^*c*^
Randomization			
Delayed FAP-testing – no. (%)	88 (58.3)	62 (44.9)	0.023^*a*^
Age years, median (IQR)	73.0 (62.0–79.0)	73.5 (58.8–81.0)	0.765
Female sex – no. (%)	74 (49.0)	54 (39.1)	0.091
Medical history – no. (%)			
Smoker	34 (22.5)	24 (17.4)	0.277
COPD	78 (51.7)	51 (37.0)	0.012^*a*^
Ongoing antibiotic treatment on admission	28 (18.5)	27 (19.6)	0.825
Clinical characteristics at presentation – median (IQR)			
CURB65	1.0 (1.0–2.0)	1.0 (1.0–2.0)	0.660
PSI	85.0 (66.3–105.0)	86.0 (65.0–108.0)	0.838
Highest value of inflammation markers during admission – median (IQR)			
WBC count ×10^9^ /L	13.3 (9.8–16.8)	10.9 (8.1–14.1)	<0.001^*a*^
CRP mg/L	138.5 (61.0–240.0)	116.0 (42.5–182.25)	0.005^*a*^
PCT µg/L	0.21 (0.00–0.63)	0.16 (0.00–0.38)	0.268
Discharge diagnosis – no. (%)			
Pneumonia	101 (66.9)	91 (65.9)	0.865
Infectious exacerbation of COPD	25 (16.6)	17 (12.3)	0.307
Non-infectious exacerbation of COPD	8 (5.3)	9 (6.5)	0.659
Other RTIs	17 (11.3)	21 (15.2)	0.320

^*a*^ Significant at *P* ≤ 0.05 (two-tailed).

^*b*^ As assessed by microscopy screening. Samples with <10 squamous epithelial cells (SECs) or a ratio of leukocytes/SECs ≥10 together with >5 microbes per field at a 100× magnification were deemed as high quality.

^*c*^ *P* values calculated by the Pearson's χ^2^ test for categorical variables and Mann-Whitney U Test for continuous variables. Not normally distributed variables were log transformed. A two-tailed *P*-value ≤ 0.05 was considered statistically significant for all analyses.

^*d*^ IQR, interquartile range; COPD, chronic obstructive pulmonary disease; CURB65, CURB-65 Score for Pneumonia Severity; PSI, Pneumonia Severity Index for CAP; WBC, white blood cells; CRP, C reactive protein; PCT, procalcitonin; RTI, respiratory tract infection.

### Bacterial detections

Bacterial detections by FAP *plus* in patients with LRT infections are provided in Table 2. Detections of bacteria were found in 95 (75.4%) high-quality samples, compared to 65 (60.2%) detections in low-quality samples (*P* = 0.013). Additionally, detections of bacteria deemed clinically relevant by predefined criteria were found in 88 (69.8%) high-quality samples compared to 59 (54.6%) in low-quality samples (*P* = 0.016). We also found that high-quality samples had more detections of *H. influenzae* (45 vs 22, *P* = 0.010). Furthermore, high-quality samples had more detections of bacteria with high semi-quantitative values, 88 (69.8%) samples with detections of ≥10^5^ copies/mL [compared to 57 (52.8%) in low-quality samples, *P* = 0.007], 81 (64.3%) samples with detections of ≥10^6^ copies/mL [compared to 43 (39.8%) in low-quality samples, *P* < 0.001], and 71 (56.3%) samples with detections of ≥10^7^ copies/mL [compared to 27 (25.0%) in low-quality samples, *P* < 0.001].

**TABLE 2:** Bacterial detections by FAP *plus* in patients with LRTI (*n* = 234)^*d*^

Detections by FAP plus	High-quality LRT samples (*n* = 126) *N* (%)	Low-quality LRT samples (*n* = 108) *N* (%)	*P*-value^*b*^	Percentage of detections that would have been missed if only high-quality samples were analyzed—% (95% CI)^*c*^
Samples with any bacterial species detected	95 (75.4)	65 (60.2)	0.013^*a*^	40.6 (33.3–48.4)
Samples with a single species of bacteria	58 (46.0)	42 (38.9)	0.271	42.0 (32.8–51.8)
Samples with a detection deemed clinically relevant and treated by the clinician	32 (25.4)	23 (21.3)	0.464	42.8 (29.7–55.0)
Samples with a bacterial detection deemed clinically relevant by predefined criteria	88 (69.8)	59 (54.6)	0.016^*a*^	40.1 (32.6–48.2)
Samples with detection of a proven etiology by predefined criteria	9 (7.1)	6 (5.6)	1.000	40.0 (19.8–64.3)
Samples with probable etiology by predefined criteria	79 (62.7)	53 (49.1)	0.036^*a*^	40.2 (32.2–48.7)
Number of bacterial species detected by FAP plus (mean per sample)	146 (1.16)	99 (0.92)	0.053	40.4 (34.5–46.7)
Detections of bacterial pathogens				
*H. influenzae*	45 (35.7)	22 (20.4)	0.010^*a*^	32.8 (22.8–44.8)
*S. pneumoniae*	25 (19.8)	17 (15.7)	0.415	40.5 (27.0–55.5)
*S. aureus*	22 (17.5)	16 (14.8)	0.584	42.1 (27.8–57.8)
*M. catarrhalis*	22 (17.5)	13 (12.0)	0.246	37.1 (23.1–53.7)
Samples with ≥10^4^ copies/mL	95 (75.4)	65 (60.2)	0.013^*a*^	40.6 (33.3–48.4)
Samples with ≥10^5^ copies/mL	88 (69.8)	57 (52.8)	0.007^*a*^	39.3 (31.7–47.4)
Samples with ≥10^6^ copies/mL	81 (64.3)	43 (39.8)	<0.001^*a*^	34.7 (26.9–43.4)
Samples with ≥10^7^ copies/mL	71 (56.3)	27 (25.0)	<0.001^*a*^	27.6 (19.6–37.1)

^*a*^ Significant at *P* ≤ 0.05 (two-tailed).

^*b*^ Pearson’s χ^2^ test for categorical variables with expected counts >5, Fisher’s exact test for categorical variables with expected counts ≤5, and independent sample *t* test for continuous variables. A two-tailed *P*-value ≤ 0.05 was considered statistically significant.

^*c*^ Confidence intervals computed by the modified Wald method.

^*d*^ FAP plus, BioFire FilmArray Pneumonia Panel plus (bioMérieux S.A., Marcy-l'Etoile, France); LRTI, lower respiratory tract infection; CAP, community-acquired pneumonia; LRT, lower respiratory tract; CI, confidence interval.

No significant differences were detected in the mean number of detections per sample, proportions of samples with a single bacterial species, samples with a bacteria deemed clinically relevant and treated by the clinician, samples with detection of a proven etiology of CAP by predefined criteria, in the number of bacterial species detected, or for the detection of *S. pneumoniae*, *M. catarrhalis*, or *S. aureus* or for any of the other bacterial targets on the panel except for *H. influenzae* between high- and low-quality samples. Detection rates for other species than *H. influenzae, S. pneumoniae*, *M. catarrhalis*, and *S. aureus* are presented in Table S1.

The results showed that 40.4% of the bacterial detections would have been missed if only high-quality samples were analyzed. These included 59 (40.1%) samples with detections deemed clinically relevant by predefined criteria, 23 (42.8%) samples with detection of a bacteria deemed clinically relevant and was treated by the treating physician, 17 detections (40.5%) of *S. pneumoniae*, 22 detections (32.8%) of *H. influenzae*, 13 detections (37.1%) of *M. catarrhalis*, and 16 detections (42.1%) of *S. aureus*.

Regarding sputum culture, 83 bacterial species were reported from high-quality samples, whereas only eight were reported from low-quality samples (*P* < 0.0001). The bacterial species identified in the low-quality samples included one *S. pneumoniae*, one *Streptococcus agalactiae*, four *H. influenzae*, one *M. catarrhalis*, and one *Streptococcus dysgalactiae*. Normal respiratory flora was grown in 105 (83.3 %) from the high-quality samples and in 105 (98.1 %) of the low-quality samples (*P* < 0.001). For a detailed comparison of detections of typical bacteria in high-quality sputum samples by culture and FAP *plus*, please refer to Table S2. This table provides comprehensive information on the concordance and discrepancies between the two methods for bacterial detections.

Fifteen patients had a proven microbial etiology of CAP as detected in their sputum sample. Nine patients had a detection of *S. pneumoniae* in their sputum sample and a positive pneumococcal urinary antigen, one patient had *L. pneumophila* detected in the sputum sample and a positive Legionella urine antigen test. Five patients had a detection of the same bacterial species in the sputum sample and in their corresponding blood cultures. For these fifteen patients, nine (60%) sputum samples were deemed as high quality and six (40%) as low quality.

## DISCUSSION

To our knowledge, this is the first study to assess the value of microscopy quality assessment of sputum samples assayed by rapid syndromic PCR-based testing. In this prospective study, we found that high-quality samples had a higher proportion of bacterial detections by FAP *plus* compared to low-quality samples, including a higher proportion of detections of bacteria deemed clinically relevant by predefined criteria and a higher proportion of detections of *H. influenzae*. However, we found no significant differences in the proportion of sputum samples with the detection of clinically important species such as *S. pneumoniae* and *M. catarrhalis* between high- and low-quality samples. Furthermore, we found no significant differences in detections, which were deemed clinically relevant and subsequently treated by the clinician, between the two groups. The results also showed that a significant proportion of detections, between 28% and 43% of the detections depending on what definition is used, would have been missed if only high-quality samples were analyzed, highlighting the importance of also analyzing low-quality samples.

Our findings are consistent with previous studies on the culture of low-quality sputum samples. One frequently cited article by Murray and Washington compared the culture of sputum samples with different qualities and transtracheal aspirates (TTA) (23). The mean number of isolates was 4.2–4.4 in low-quality sputum samples, 2.7 in high-quality sputum samples, and 2.4 in TTAs. However, the most frequently detected species were viridans group streptococci, *Staphylococcus epidermidis*, *Neisseria* spp., *Haemophilus* spp. other than *H. influenzae*, Yeast, and *Corynebacterium*. Common respiratory pathogens such as *S. pneumoniae*, *H. influenzae*, *Moraxella* spp., and *S. aureus* were only detected in 0.5%, 3.3%, 3.3%, and 12.1% of low-quality samples, respectively. When looking at only species likely to cause pneumonia, there were not more isolates in low-quality sputum samples than in TTAs and more detections in the high-quality samples (26). The total number of detections of *H. influenzae* is not available in the article. For *S. pneumoniae*, *Moraxella* spp., and *S. aureus* 41%, 36%, and 47% of detections were in low-quality sputum samples. In another article by Geckler et al. compared 96 sputum samples from patients with pneumonia to TTAs, with the majority of patients being young adults with no significant underlying diseases (27). The study considered *S. pneumoniae*, *H. influenzae*, *Neisseria meningitides*, *S. pyogenes*, *S. aureus*, and enteric gram-negative rods as pathogens. The study found that 41% of TTAs had more than 10 SECs per field. The false positive rate was 36% in low-quality sputum samples and 9% in high-quality sputum samples. However, they examined only 11 low-quality sputum samples. Wong et al. examined 391 unselected sputum specimens (6). Potential pathogens were recovered from 143 specimens. Between 9% and 34% of specimens with potential pathogens would have been rejected depending on what microscopy criteria were used. In a more recent study, Saukkoriipi et al. studied sputum quality assessment for the culture of respiratory specimens, albeit only for *S. pneumonia* from elderly CAP patients (8). They found that the culture of low-quality sputum samples had lower sensitivity than high-quality samples, but the specificities were the same. Additionally, 35% of encapsulated pneumococci were detected in low-quality sputum samples for all patients and in 31% of patients with *S. pneumoniae* verified in blood cultures. Encapsulated pneumococci were cultured at similar proportions in high- and low-quality sputa, if another pneumococcal test such as blood culture, urine antigen test or a twofold increase in pneumococcal antibodies were concomitantly positive (8). When considered alongside previous literature, our study indicates that the sensitivity of microscopy falls short of modern screening test requirements (28).

Microbiological analysis of respiratory samples based on traditional culture methods is labor-intensive, involving a series of complex manual handling steps, that necessitate a high level of training for technologists. Many of the arguments related to the laborious workflow in processing respiratory samples are not relevant for syndromic PCR-based testing, and moreover are not affected by the additional presence of oral microbiota in the samples. The fact that a significant difference was found in the proportion of samples with high semi-quantitative values (≥10^4^ copies/mL by FAP *plus*) in high-quality samples compared to corresponding values in low-quality samples is interesting, as previous studies have reported relatively low (40%–54%) concordance between FAP *plus* semi-quantitation and sputum culture (14, 29, 30). However, previous studies on sputum culture have found a level of detection of 10^5^ to 10^8^ for the detection of bacteria in Gram stains (31). The cut-off values for semi-quantitative multiplex PCR have been determined by expert opinion and are based on the assumption that organisms detected in greater quantities are more likely to be clinically significant. However, given the lack of suitable comparator “gold” standards, these cut-off values are difficult to corroborate and may also vary for different microorganisms (32). Moreover, other studies that have included the FAP *plus* have questioned the relevance of detections of 10^4^ and even 10^5^ copies/mL based on low positive agreement compared to LRT sample culture (33, 34).

A considerable strength of our study is the generalizability of the results to a large group of hospitalized patients with LRT infections. This is ensured by the broad inclusion criteria as all adult patients, except for those with cystic fibrosis (*n* = 8) or receiving palliative treatment (*n* = 3) presenting to the ED with suspected CAP and who were able to consent and/or co-operate to sputum collection were eligible for inclusion. Sputum samples were collected by trained study nurses or physicians in the ED. Our study population’s age and comorbidity distribution are comparable to those reported in larger CAP studies (35, 36).

False positive detections of potential pathogenic bacteria are a concern when interpreting low-quality sputum samples (37), although this assumption is not supported by either our study or previous studies (8, 23). As with all diagnostic tests, test performance is heavily influenced by the pretest probability of the disease (38). In our study, we only included patients hospitalized with confirmed LRT infections in the analysis of bacterial detections, making the detection of potential pathogenic bacteria in this patient population significantly more likely to be clinically relevant than in a patient without a certain LRT infection.

Other strengths include: first, only mucoid and purulent and no visually saliva-like samples were submitted for testing, and second, all samples were analyzed by the FAP *plus* assay which provided an objective semi-quantitative result. While culture may fail to accurately recover all pathogens from a complex matrix and is influenced by subjective interpretation, the FAP *plus* is more robust against such inherent variability (14).

This study has limitations. There is no established “gold standard” for determining the etiology of pneumonia. Sputum harvested in our study has the probability of contamination by upper respiratory tract flora, a feature that is inherent to all non-invasive sampling techniques. The ideal method for sampling is by obtaining samples directly from the site of infection without passing the upper airways, such as pleural fluid sampling, percutaneous fine needle aspiration, or lung biopsy (39). However, these invasive procedures are rarely performed in clinical practice. In the current study, we used a pragmatic approach and compared the detections of possible microbial etiologic agents of CAP in high- and low-quality sputum samples. A consistent finding was that a large proportion (28%–42%) of the detections would have been missed if only high-quality samples were analyzed. This was also true for CAP cases with proven microbial etiology, suggesting that our findings are representative.

For both FAP *plus* results communicated to the treating physician and for interpretation of culture results by technologists, the results from the initial microscopy sputum evaluation were readily available. Ideally, this information should have been withheld; however, blinding microscopy results was not a part of our RCT protocol. Nevertheless, the treating physicians still considered 24 (42%) detections in low-quality samples clinically relevant.

For both FAP *plus* results and culture assays, the treating physician and the laboratory technologists, respectively, were not blinded to the results from the initial microscopy evaluation. Ideally, they should have been; however, blinding of microscopy results was not a part of the RCT (NCT04660084) protocol. However, the treating physicians still considered 42% of the bacterial detections in low-quality samples as clinically relevant. The fact that laboratory technologists were not blinded to the microscopy results is probably reflected in the large difference in the amount of reported bacterial species in low-quality samples from culture.

There are also limitations to all culture-independent methods that can limit their applicability in clinical practice. Multiplex PCR assays cannot accurately distinguish DNA from viable vs non-viable organisms. Detection might represent historic infections without a need to treat (40). Moreover, the lack of detection of off-target pathogens, a lack of full susceptibility information, and cost are additional limitations to be considered. However, notably neither culture nor Nucleic Acid Amplification Tests separates airway colonizers from invasive pathogens.

This investigation was conducted as part of a larger RCT (NCT04660084), which aimed to examine the effects of syndromic PCR testing with the FilmArray Pneumonia Panel Plus (FAP Plus) on antimicrobial use. In the parent trial, patients were randomized into two arms: one receiving FAP Plus testing in addition to standard of care diagnostics and the other receiving standard of care diagnostics alone. Although the randomization process in the parent trial was not directly related to the specific research questions addressed in this study, we included it to provide context and clarify the source of the data used for the analysis. It is important to note that the results from FAP Plus were not available to clinicians for approximately half of the patients, which may have influenced treatment decisions in those cases. To our knowledge, no previous study has shown a clinical benefit of microscopy quality assessment of sputum samples. Assessment of sputum quality is based on tradition and on studies aimed at optimizing the laboratory workflow, with most studies conducted more than 30 years ago. These studies did not provide relevant patient characteristics, including age and comorbidity status of the patients included (6, 23, 24, 41, 42), or were studies where samples were collected from young adults with no significant underlying diseases (27). It is therefore highly speculative whether the inference made from these earlier studies applies to the current population admitted to hospitals with pneumonia, with a predominance of elderly persons with comorbid illnesses (43). Notably, the challenge with the culture of low-quality samples is low sensitivity, not low specificity or high false positive rates (23, 27, 44).

The findings of our study suggest that the current microscopy criteria for evaluating sputum quality are rigid and harbor the risk of excluding isolates that are deemed clinically relevant. We question the rejection of sputum samples for rapid syndromic PCR testing based on an initial microscopy quality assessment and recommend analysis by PCR-based tests and/or culture of all visually non-serous sputum samples and suggest that the prerequisite of initial microscopy-based quality assessment of respiratory samples be re-considered.

## SUPPLEMENTAL MATERIAL

Supplemental tables spectrum.03002-23-s0001.pdf Tables S1 and S2.

ASM does not own the copyrights to Supplemental Material that may be linked to, or accessed through, an article. The authors have granted ASM a non-exclusive, world-wide license to publish the Supplemental Material files. Please contact the corresponding author directly for reuse.
