# Supplementary material for: The diagnostic utility of microscopic quality assessment of sputum samples in the era of rapid syndromic PCR testing
Source: Microbiol Spectr. 2023 Sep 29;11(5):e03002-23. doi: 10.1128/spectrum.03002-23 (PMC10581175; doi:10.1128/spectrum.03002-23)
Supplement: Supplemental tables — Tables S1 and S2. [file spectrum.03002-23-s0001.pdf]

Supplementary material

Supplementary table 1: Detections of typical bacteria sorted by number of detections

**Detections by FAP *plus***	**High quality LRT samples** (n=126) **N (%)**	**Low quality LRT samples** (n=108) **N (%)**	**p-value[1]**	**Percentage of detections that would have been missed if only high quality samples were analysed - % (95 % CI[2])**
Haemophilus influenzae	*45 (35.7)*	*22 (20.4)*	*0.010**	*32.8 (22.8-44.8)*
Streptococcus pneumoniae	25 (19.8)	17 (15.7)	0.415	40.5 (27.0-55.5)
Staphylococcus aureus	22 (17.5)	16 (14.8)	0.584	42.1 (27.8-57.8)
Moraxella catarrhalis	22 (17.5)	13 (12.0)	0.246	37.1 (23.1-53.7)
Escherichia coli	8 (6.3)	6 (5.6)	1.000	42.9 (21.3-67.5)
Pseudomonas aeruginosa	8 (6.3)	5 (4.6)	0.776	38.5 (17.6-64.6)
Streptococcus agalactiae	2 (1.6)	7 (6.5)	0.085	77.8 (44.3-94.7)
Klebsiella pneumoniae group	5 (4.0)	2 (1.9)	0.456	28.6 (7.6-64.8)
Proteus spp.	1 (0.8)	4 (3.7)	0.184	80.0 (36.0-98.0)
Serratia marcescens	4 (3.2)	1 (0.9)	0.377	20.0 (2.0-64.0)
Enterobacter cloacae complex	2 (1.6)	2 (1.9)	1.000	50.0 (15.0-85.0)
Klebsiella oxytoca	1 (0.8)	3 (2.8)	0.338	75.0 (28.9-96.6)
Acinetobacter baumanii complex	1 (0.8)	0	1.000	0.0 (0.0-83.3)
Klebsiella aerogenes	0 (0)	0 (0)	NA	NA
Streptococcus pyogenes	0 (0)	0 (0)	NA	NA

*Significant

[1]Pearson's chi-squared test for categorical variables with expected counts >5, Fisher's exact test for categorical variables with expected counts ≤5, and independent sample t test for continuous variables. A two tailed p-value ≤ 0.05 was considered statistically significant.

[2]Confidence intervals computed by the modified Wald method

Abbreviations: FAP plus - BioFire® FilmArray® Pneumonia Panel plus (bioMérieux S.A., Marcy-l'Etoile, France; LRT - lower respiratory tract; CI - confidence interval

Supplementary table 2: Detections of typical bacteria in high-quality sputum samples by culture and FilmArray pneumonia panel *plus* (FAP *plus*) in 126 patents with pneumonia or infectious exacerbations of chronic obstructive pulmonary disease

	**Not detected**<br><br>**N**	**Detected by FAP *plus* only**<br><br>**N (%)**	**Detected by culture only**<br><br>**N (%)**	**Detected by both culture and FAP *plus***<br><br>**N (%)**
Acinetobacter baumanii complex	125	1 (100)	0 (0)	0 (0)
Enterobacter cloacae complex	124	2 (100)	0 (0)	0 (0)
Escherichia coli	118	1 (12.5)	0 (0)	7 (87.5)
Haemophilus influenzae	79	10 (21.3)	2 (4.3)	35 (74.5)
Klebsiella aerogenes	126	0 (NA)	0 (NA)	0 (NA)
Klebsiella oxytoca	124	0 (0)	1 (50)	1 (50)
Klebsiella pneumoniae	120	4 (66.7)	1 (16.7)	1 (16.7)
Moraxella catarrhalis	102	9 (37.5)	2 (8.3)	13 (54.2)
Proteus spp	125	1 (100)	0 (0)	0 (0)
Pseudomonas aeruginosa	117	2 (22.2)	1 (11.1)	6 (66.7)
Serratia marcescens	122	2 (50)	0 (0)	2 (50)
Staphylococcus aureus	103	15 (65.2)	1 (4.3)	7 (30.4)
Streptococcus agalactiae	124	2 (100)	0 (0)	0 (0)
Streptococcus pneumoniae	100	9 (34.6)	1 (3.8)	16 (61.5)
Streptococcus pyogenes	126	0 (NA)	0 (NA)	0 (NA)

Abbreviations: FAP plus - BioFire® FilmArray® Pneumonia Panel plus (bioMérieux S.A., Marcy-l'Etoile, France
